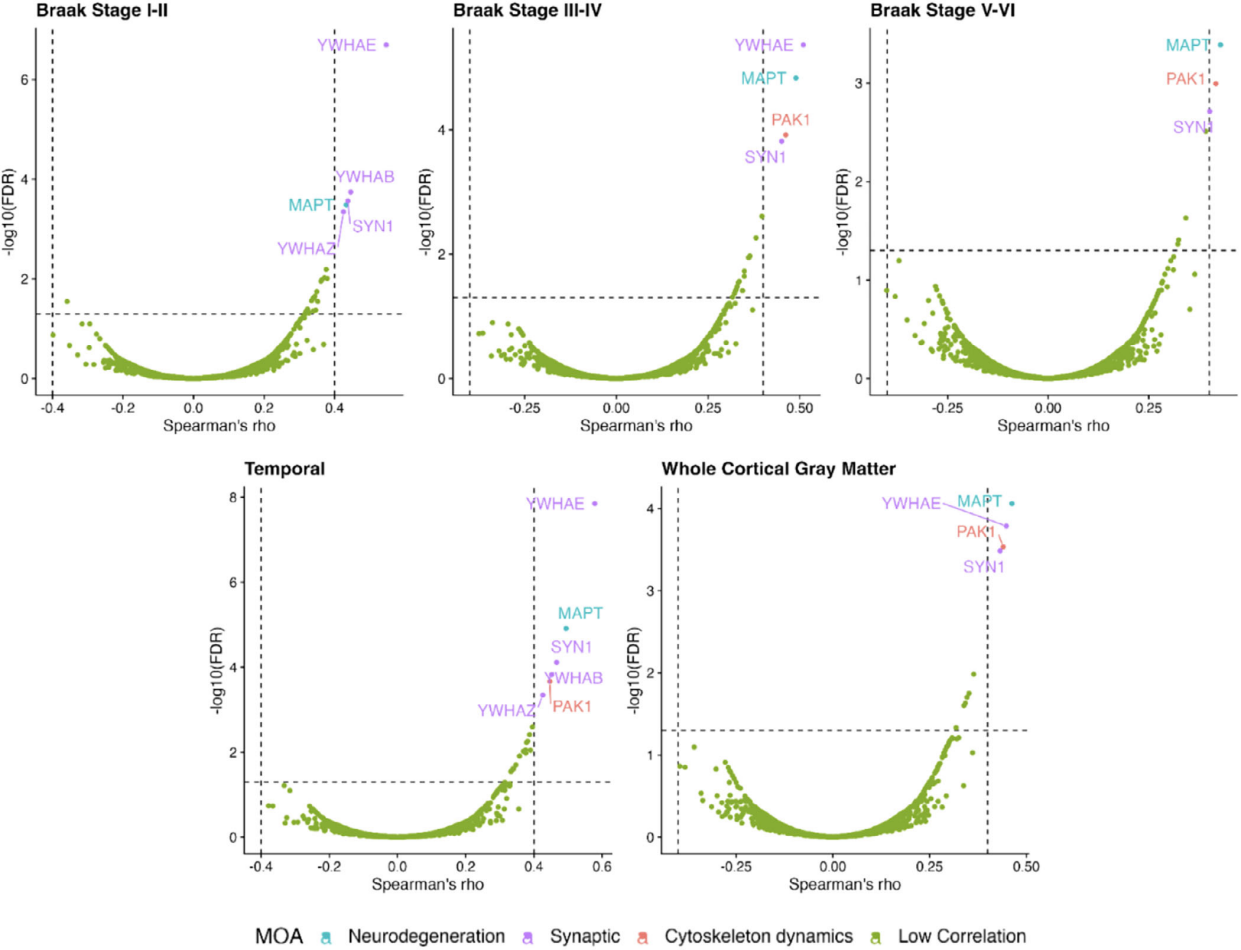# Identification of CSF proteins in Alzheimer’s Disease highly correlated with [^18^F]GTP1

**DOI:** 10.1002/alz.089230

**Published:** 2025-01-09

**Authors:** Julie Lee

**Affiliations:** ^1^ Genentech, South San Francisco, CA USA

## Abstract

**Background:**

Neurofibrillary tangles (NFTs) are neuropathological hallmarks of Alzheimer’s Disease (AD) and consist of insoluble aggregates of tau protein (MAPT). Tau pathology spreads in the brain with increasing disease severity. Tau is commonly measured in AD trials as it is diagnostic and prognostic of clinical progression, and also a pharmacodynamic biomarker to quantify treatment effects of emerging tau‐targeting therapeutics. Tau PET tracers, like [^18^F]Genentech Tau Probe 1 ([^18^F]GTP1), selectively bind to NFTs to enable imaging of tau pathology in AD patients. Tau PET imaging requires special infrastructure at clinical sites and has high costs limiting its broad applicability in trials. The goal of this analysis is to identify CSF proteins highly correlated with [^18^F]GTP1 to develop fluid surrogate biomarkers of tau PET that may allow greater accessibility to pathological tau measurements in clinical settings.

**Method:**

AD CSF samples were from participants enrolled in Tauriel or Lauriet, two semorinemab PhII trials. Baseline protein levels were measured by analyzing samples using data‐independent acquisition mass spectrometry (DIA‐MS). After data processing and filtering, 3197 unique protein intensities were included in this analysis. Trial participants also completed [^18^F]GTP1 imaging. Analysis was completed by Invicro where standardized uptake value ratios (SUVR) were reported using inferior cerebellar gray matter as the reference area. The five regions evaluated in this analysis are whole cortical gray matter, temporal, Braak stages I‐II, Braak stages III‐IV, and Braak stages V‐VI. [^18^F]GTP1 SUVR values for the selected regions were compared against CSF protein intensity measurements to calculate Spearman correlation coefficients and FDR‐adjusted p‐values to identify highly correlated proteins.

**Result:**

CSF proteomic measurements and [^18^F]GTP1 SUVR values were available from 128 Tauriel or Lauriet participants at baseline. The correlation analysis demonstrated proteins MAPT, SYN1, PAK1, YWHAE, YWHAB, and YWHAZ are highly correlated with [^18^F]GTP1 across more than one brain region. Proteins with absolute correlation values greater than 0.4 with tau PET are functionally related to the synapse.

**Conclusion:**

Six CSF proteins highly correlated with tau PET were identified. Validation of these results by replicating the analysis on a secondary dataset with PET and CSF protein measurements may establish further confidence in these findings.